# Abstracts and Gleanings

**Published:** 1879-06-20

**Authors:** 


**Chloroform in Labor.**—Prof. Courty says: "In employing chloroform for lying-in women I observe the same precautions as in all my operations. In place of having her chloroformed by an assistant, so as to produce both insensibility and muscular relaxation, assimilating her to a corpse, as I have sometimes seen done in England on patients about to be operated upon (which I may say, in passing, explains to me the incomparably greater frequency of deaths from chloroform in that country compared with our own), I cause her to breathe the chloroform by little whiffs, and mixed at first with plenty of air, making her count aloud, in order to cause her to respire regularly, and at the same time render me an account of the condition of the nervous centres. I suspend the anæsthesia when the pains have been rendered tolerable, resuming it and suspending it again according to the necessity. I have thus, without any danger, been able to prolong anæsthesia in women in labor from one hour to eight and even ten hours, and have consumed in a day, in small whiffs 120, and perhaps even 150, grammes of chloroform. I say, 'perhaps,' for it is difficult to dose it when the most simple and least frightening mode of administration is employed, viz., by a sponge placed in a curved napkin. Thus used, so little fear does it excite, so easy is it employed, and so well tolerated by most patients, that they familiarize themselves with it to the risk of converting it into an abuse if not carefully watched."—*Med. Times and Gazette.*

**Sodium Ethylate in Nævus.**—This substance is prepared by adding metal sodium, piece by piece, to absolute alcohol, in a wide-mouthed bottle, until effervescence ceases, when a deposition of a crystalline substance occurs. The clear liquid is the part used. It is a potent caustic, causes less pain and scarring than nitric acid, and has been very successfully used for removing nævi.—*New York Med. and Surgical Journal.*

### ABSTRACTS AND GLEANINGS.

**Lactopeptine.**—As digestion is the most complex of all organic processes, its derangements, which constitute indigestion or dyspepsia, are the most complicated of all morbid conditions. The profession have doubtless frequently observed how intimate are the connections between the progress of disease and the consequent derangements that invariably follow in the digestive system, not to appreciate fully the value of a medicinal agent which secures the perfect assimilation of the remedies indicated, and enables the patient to thoroughly digest the nutriment taken.

A reference to the formula of lactopeptine will show at once what valuable results must necessarily follow its administration in all cases of mal-nutrition and non-assimilation. Composed of pepsin, pancreatine, ptyalin, lactic and hydrochloric acids, it is a combination of all the digestive agents that are known to exist in the human system, and can therefore never be administered in the foregoing cases without yielding the most satisfactory results.

From our personal experience with lactopeptine, in cholera infantum, vomiting in pregnancy, in the several forms of dyspepsia, and in chronic diarrhœa, we can most unhesitatingly pronounce it to be a remedy of the greatest importance, and fully justifying the remark made to us by a physician of this city, that he considers the eminent pharmacists who had introduced lactopeptine to our profession should hereafter always be numbered among the benefactors of mankind.—*Ex.*

**Bronchitis from Mouth Breathing.**—Dr. Milner Fothergill, in Medical Times, writes: When air is respired through the nostrils it is heated by the warm plates of the turbinated bones, with their rich vascular supply over them in their mucous membrane. But these poor children crying and moaning breathe almost exclusively by the mouth and but little by the nostrils, which are more or less plugged with mucus, that the little custodians are not watchful in removing. Consequently they inspire the cold air, and the residual air in the lungs becomes persistently chilled, and then hyperæmia of the bronchial lining membrane follows, and runs on into inflammation. The struggle for life is very brief with these ill-nourished infants; the respiratory centre cannot carry on a long fight, the bronchiæ become choked with phlegm, which the organism is unequal to removing, and then the life flickers out. It is, unfortunately, quite impossible to see how such disease is to be prevented at least in the present state of our knowledge. As to treatment, it must be as energetic as circumsances will permit; and I am inclined to think that the effects of temperature on the centres of the circulation and the respiration are not sufficiently attended to. The effect of a low temperature is to lessen the activity of these two centres; while heat stimulates them. Consequently, when the body-temperature falls, these centres are partially paralyzed, and the advantage to be derived from hot drinks and the jacket poultice are obvious. They greatly aid the action of direct stimulants to those centres, as ammonia, strychnia, beladonna, etc. The efficacy of these means was well illustrated in a case where the writer was called in consultation lately. A lady, who was liable to attacks of asthma, was severely ill with bronchitis. Her pulse was 150, and respirations 40 per minute. The respiratory act being labored, the face was dusky, the temperature 102°, though the perspiration was profuse. The only scintilla of hope lay in the fact that the right ventricle might have been trained by the attacks of asthma to stand a long strain. Ammonia and strychnia with spirits of chloroform were given, whisky with milk ordered freely, and the chest was encased in linseed meal poultices. She rallied, and made an excellent if scarcely hoped-for recovery.

**Spinal Curvatures.**—Dr. Macleod tried other substances besides plaster of Paris, such as paraffin, glue, starch. Glue did pretty well, but was not equal to plaster; while paraffin did not do well, and was dirty to handle. He also pointed out that instead of Sayre's suspension apparatus, it was easy to improvise with a room door an arrangement that would serve the purpose. As regarded abscesses, which sometimes occurred in Pott's diease, Sayre, who did not believe in antiseptic surgery, opened them freely, and cleansed out the abscess with Peruvian balsam (an antiseptic). Dr. Macleod then demonstrated minutely the further treatment for abscesses. He also shewed by means of a model, that as proved by Sayre, in what was usually called lateral curvature, there was a rotation of the bodies of the vertebræ upon themselves. On this account he (Sayre) had substituted the term "Rotary Lateral" for lateral, as being descriptive of the exact state of matters. In regard to this kind of curvature, all the ordinary kinds of apparatus went on a wrong principle, and did harm. The object was to get back muscular tone, and this was done by exercising the muscules which had lost their energy. Mere lateral pressure would do no good at all. The spine must be straightened by self-suspension several times daily, for months at a time. The hand on the concave side should be held uppermost. After a considerable experience of these cases of curvature, he had no hesitation in saying that Sayre's treatment of them was very far in advance of any former methods of treatment which he had tried.—*Glasgow Med. Journal.*

**Tubercular Ulcer of the Tongue.**—M. Nedopil, in the Archiv. for Klinische Chirurgie, remarks that the diagnosis of secondary tubercular ulcer of the tongue is generally not difficult in the presence of other indications of tuberculosis. On the other hand, primary tubercular ulcer can often be scarcely distinguished from cancer unless a microscopic examination be made; while the failure of anti-syphilitic treatment distinguishes it from syphilitic ulcer, which often has a similar appearance. The tubercular ulcer of the tongue runs a course resembling that of cancer. A small hard nodule on the edge or upper surface of the tongue, which is often overlooked, at last falls off, and leaves a dirty ulcer, with an indurated base, which generally spreads more slowly than a cancerous ulcer. A cure can be produced only by early extirpation, which, perhaps, may arrest the development of general tuberculosis. The author has observed four cases in Billroth's clinic; two of the individuals were thirty-two years of age, the others sixty-eight and seventy. In three cases the ulcer was extirpated, and healing took place in a few days. In the exercised pieces the tissue around the ulcer was studded with miliary tubercles, mostly toward the free surface. The morbid process appears to commence with a general transformation of the muscular tissue into a homogeneous slightly granular deposit containing proliferating muscle-nuclei. Later, the primary deposits become confluent, and giant cells are formed from the obstructed portions of the blood-vessels; in some of these Nedopil found cavities filled with brown pigment. The growth of the tubercle appears to take place partly through proliferation of nulcei (without cell formation) in the interior, partly through metamorphosis of the neighboring tissue.—*The Doctor.*

**Rules for Injecting Piles.**—If the following rules be observed I believe that the method of treatment by hypodermic injection will be less painful than any other, and at the same time equally safe:

1. Inject internal piles only.
2. Use the more diluted forms of the remedy first, and the strong ones only in case these fail.
3. Treat one pile at a time, and allow from four to ten days between the operations.
4. Inject from one to four drops, smearing the surface with cosmoline to guard against dripping. Inject very slowly, and keep the pipe in its place a few moments to allow the fluid to fix itself in the tissues.
5. Confine the patient to bed the first day, and return him to it subsequently if any severe symptoms occur. Prohibit any but very moderate exercise during the treatment.

Piles under all treatments, as well as when left without treatment, are subjeet to possible hemorrhage. Allingham recommends the following method of applying the tampon for all cases where the bleeding vessel cannot be promptly found and controlled: He takes a pretty large sponge and fastens a strong double string through its centre. (He prefers a bell-shaped sponge inserted with the open end downward.) Having pushed the sponge up the rectum some inches beyond the bleeding-point, he fills the parts below with cotton dusted with powdered alum or persulphate of iron, and ties a stick across the finished tampon with the double string. By turning the stick around like the handle of a gimlet he twists and tightens the string, forcing the tampon firmly up against the sponge, and causing it to spread laterally and to compress the bleeding vessels. He advises to put in a large catheter with the tampon to give exit to the flatus. By the use of opiates the tampon is often tolerated several days.

My final conclusion is that the wild itinerants of the prairies have really made a valuable contribution to scientific knowledge, and that the cautious injection of hemorrhoids with carbolized solutions will remain as one of the permanent operations of surgery.—*Dr. Summers, in Lancet and Clinic.*

**Salicylate of Quinine as an Antipyretic.**—This is the subject of a short article by Dr. J. G. Brown, in the November number of the Edinburg Medical Journal. He says:

The salt is prepared by the action of salicylate of soda on sulphate of quinine. It is insoluble in water, but dissolves readily in certain acid solutions, and tolerable easy in cold, still better in hot, alcohol. In the cases to which it has been administered, the dose has ranged from ten to forty grains according to the age of the patient and the mode of administration, thirty grains in most cases being sufficient. Its antipyretic action usually begins in about an hour if it has been administered by the mouth, and continues for some hours subsequently. In some cases the temperature has fallen as much as five degrees in the course of three or four hours. Simultaneous with antipyresis the pulse becomes slower and softer, the respirations also diminish in frequency, the skin becomes cool and moist, and generally the condition of the patient is much improved. He expresses himself as cool and comfortable, and sleep usually follows.

The action of salicylate of quinine is free from the profuse perspirations which attend that of salicylate of soda; and, on the other hand, the ringing in the ears and deafness, whieh are occasioned by full doses of sulphate of quinine, are hardly noticed after the administration of salicylate of quinine, if an over-dose be not given. The vomiting which large doses of sulphate of quinine so often produce, is entirely absent after the exhibition of salicylate of quinine, nor has albuminuria ever been noticed.

With regard to the mode of its administration, it may be given by the mouth, either dry (in wafer paper), or suspended in mucilage, or in water; but in severe cases, (especially in enteric fever), it is best given as an enema, suspended in mucilage with a small quantity of laudanum to soothe the mucous membrane of the rectum.—*The Physician and Pharmacist.*

**Vaseline and Unguentum Vaselini Plumbicum in Skin Disease.**—Prof. Kaposi, after stating that all emollient substances hitherto used in diseases of the skin, where the epidermis is removed or the surface is sensitive, as various fatty substances, oils, lard, glycerine and glycerine and starch, are more or less irritating in most cases, refers to the bland and non-irritating properties of vaseline or petroleum jelly (with this also may be classed ozokerin and unguentum petrolei). These have no tendency to become rancid, and are useful in softening and removing crusts and scales, as in cases of eczema squamosum when the surface is dry and desquamating. He introduces an ointment which promises to prove of great value. This is a modification of Hebra's well-known unguentum diachyli which is seldom met with properly prepared except at Vienna. This ointment, for which Kaposi proposes the name of unguentum vaselini plumbicum, is made by dissolving and incorporating thoroughly by aid of heat equal parts of lead plaster and vaseline, to which a little oil of bergamot may be added to scent. It causes no burning sensation on excoriated parts, and is especially available in eczema. It is admitted by Kaposi that the original unguentum diachyli gave rise to unpleasant and even acute exacerbations of the eczema, due, he believes, to an evolution of fatty acids from the oil during boiling, and to an imperfect saponification of the oxide of lead.—*Edinburg Med. Journal.*

**Rheumatism of the Diaphragm.**—Dr. Mader, in the yearly report of the Rudolf Institution in Vienna, remarks that the diagnosis of a rheumatic or neuralgic affection of the diaphragm is evidently more a matter of inference than of certain evidence. Yet from time to time cases come under observation, which scarcely admit any other explanation. In the present instance, the patient was a powerful muscular butcher, twenty-seven years of age, who was attacked one morning with very severe pain, extending from the scrobiculus cordis to the back, and greatly impeding respiration. The breathing was quick, short and superficial; purely thoracic. Movements of the abdominal walls, indicating contraction of the diaphragm, were almost entirely absent. There was much turgor of the face, but no marked febrile symptoms. Nothing abnormal was found on examining the chest. A subcutaneous injection of morphia in the epigastrium was followed by a cessation of pain and by sleep.

Next morning the patient was free from all difficulty of breathing, but he complained of pain in the right scapular region. This also was relieved by injection of morphia, and the patient was discharged cured on the third day.—*London Med. Record.*

**First Insensibility from the Inhalation of Ether.**—At a late meeting of the Therapeutical Society of New York, Dr. R. F. Weir, Chairman of the Committee on Surgical Procedure and Appliances, presented a report on the first insensibility from ether, as described by Dr. John H. Packard in the American Journal of the Medical Sciences.

Dr. Weir stated that a number of cases have been reported to the committee confirmatory of the statements of Dr. Packard, but certain differences were observed. No question as to the satisfactory degree of anæsthesia exists, but the duration of it in several instances exceeded the time allotted to it by Dr. Packard—occasionally reaching to three minutes; also, while all recollection of pain was done away with, yet at times the patients would by movement, and sometimes by cries, give evidence of sensation during the incision; and, still further, it was noticed by Dr. W. T. Bull and himself that, even when the insensibility was marked, muscular relaxation was oftimes insufficient to permit of a dislocation or a displaced fracture.

One case reported by Dr. Gibney shows that in chloroform inhalation the "first insensibility" exists—a point upon which Dr. Packard, from want of experience, was unable to speak. Dr. Weir adds that this "first insensibility," or, as it is commonly called in New York, "primary anæsthesia," has now become fully established in the practice of the New York and Roosevelt Hospitals.—*Amer. Journal of Med.*

**Remedy for Dysentery.**—In the Indian Medical Gazette for 1st October, 1878, there is an interesting account of a new remedy for dysentery "which promises to rival ipecacuanha in its power over acute dysentery." The credit of bringing this remedy to notice belongs to Assistant-Surgeon Umrito Lell Deb, attached to the Howrah General Hospital. This gentleman reports, and his report is confirmed by Surgeon-Major R. Bird, M.D., Civil Surgeon of Howrah, that the root of the plant called in Bengalee *Rungun*, belonging to the genus *Ixora*, "is very efficacious in the treatment of acute dysentery." Dr. King, Superintendent of the Calcutta Botanical Gardens, identified the plants used in the trials at Howrah as belonging to the species *I. Coccinea* and *I. Bandhuca.* It is claimed for this remedy that it has the virtues of ipecacuanha without the nauseating properties of that valuable drug. At Howrah the remedy was used in doses of from fifteen to thirty grains three or four times a day, of the fresh root ground to pulp on a "curry stone," with a piece of the long pepper, administered suspended in water. Extensive trials are now being made in India of this new remedy. A tincture has also been prepared of the fresh root. —*British Medical Journal.*

**Oxide of Zinc in Diarrhœa.**—Dr. Jacquier has followed, in the service of Dr. Bonamy at Nantes, the good effects of the employment of oxide of zinc in diarrhœa. The formula which he has employed is the following:

| | | |
|---|---|---|
| ℞ | Oxide of zinc.................................... | gr. 54. |
| | Bicarbonate of soda.......................... | gr. 7½. |

in four packets, one to be taken every six hours.

In all the cases which he observed, oxide of zinc produced rapid cure of diarrhœa. In fourteen cases observed by Puygautier, the cure was even more rapid, since in only one case were three doses of medicine required. The results are considered to have been more satisfactory, inasmuch as in several cases the malady had endured from one to many months, and other methods of treatment had not produced any improvement. Thus he concludes that, although by no means to be held as exclusive treatment, the employment of oxide of zinc deserves to be more generally known as useful in diarrhœa.—*British Med. Journal.*

**Treatment of Infantile Paralysis.**—During the acute or febrile stage, Dr. Simon of Paris, recommends the use of the vapor bath, administered to the patient in bed, diaphoretics and soothing draughts, with dry cupping or fly blisters to the vertebral column. When the disease has entered on its second stage, and the paralysis is localized, frictions and kneading. Electricity should also be persevered in; the continuous current is best, very weak, and applied from above downward, with precaution. At the same time, fresh air, sea or sulphur baths, quinine, cod-liver oil in the winter, and arsenic in the summer. Gymnastic exercises for the affected limb. In fact, for this stage of the malady the various means calculated to stimulate the muscles or the nervous system should be employed sucessively or in combination. In the third stage, when deformity has occurred, recourse must be had to orthopedic apparatus. Dr. Simon rejects tenotomy absolutely in cases of paralytic club foot, the only result of the operation being to do away with the action of the only muscles which can serve the child as support.—*Medical and Surgical Reporter.*

**Epithelioma.**—Dr. Duhring, in Penn. Hospital, remarked: Epithelioma, as a rule, was found on older persons. The affection belonged to the flat or superficial variety of epithelioma, and the treatment appropriate to the case was cauterization by means of caustic potassa. The knife was not called for in this instance. The solid stick of potassa fusa was then applied, going slowly over every portion of the diseased structure, and allowing the influence of the caustic to penetrate a little below and beyond the diseased tissue in every direction. It was not necessary, Dr. D. said, to use any pressure or force, but the caustic should be brought most carefully in contact with every particle of the growth, lest recurrence should take place, and a second operation be required. When the action of the caustic had gone far enough, it was limited and checked by the application of dilute acetic acid. The pain of the operation was quite sharp and severe, but ceased almost immediately upon the application of the acetic acid. The patient was directed to dress the part simply with lint soaked in olive oil; the dressing to be removed twice daily and the wound cleansed.—*Medical and Surgical Reporter.*

**Fish-Bones in the Pharynx.**—When a fish-bone gets lodged in the throat, Prof. Voltolini thinks the worst thing a physician unpracticed with the laryngoscope can do is, to try to push the foreign body down into the stomach. This manœuvre never succeeds, but simply wedges the bone tightly in between the folds of the mucous membrane. Voltolini, therefore, suggests under such circumstances to let the fish-bone alone until a physician be found able to extract it under guidance of the throat mirror. But in the meantime, he advises the use of diluted hydrochloric acid as a gargle. He found, in a solution of 4.0 hydrochloric, or nitric acid, 1 to 240.0 water, the hardest fish bone becomes soft and flexible in one hour; thinner bones were like threads after one-half hour. And he thinks the continued gargling with the above mixture (the teeth being protected by oil or lard) will have the same effect upon a fish-bone lodged in the pharynx.—(*Monatsschrift f. Ohrenheilkt*)—*Chicago Med. Examiner.*

**A Forerunner of Death.**—Dr. Chiappelli says, in Lo Sperimentale, that he has frequently noticed in patients who were apparently very far from death an extraordinary opening of the eyelids, so as to give the eyes the appearance of protruding from the orbits, which was invariably a sign that death would occur within twenty-four hours. In some cases only one eye is wide open, while the other remains normal; here death will not follow quite so rapidly, but in about a week or so. It is easy to observe this phenomenon when the eyes are wide open; but when, as is generally the case, the eyes are half shut and only opened from time to time, it will be found advisable to fix the patient's attention upon some point or light so as to make him open his eyes, when the phenomenon will be seen. The author is utterly at a loss to explain this symptom, and ascribes it to some diseased state of the sympathetic nerve.—*British Med. Journal.*

**Remedy for Sciatica.**—Dr. Ebrard, physician to the Hospital of Nimes, publishes in the Courrier Medical this new method of treatment. For many years he has treated the pains of sciatica and other neuralgias without having recourse to any other electric battery than a smoothing iron, which, along with vinegar, is to be found in every house. This is how they are employed : The iron is heated hot enough to vaporize the vinegar, and is wrapped up in some material, preferably woollen; it is then dipped in the vinegar and applied on the painful part. The operation is repeated two or three times in the day. It rarely happens that the pain has not disappeared at the end of twenty-four hours. This action is easily understood. On account of its contact with the fire the iron becomes magnetic; and if an acid be added when it is hot, electricity is produced, and the same effects are obtained as with an electric battery.—*Canadian Journal of Med. Science.*

**Antidote to Arsenic.**—Dr. James B. McCaw, before the Richmond Academy of Medicine, remarked that dialyzed iron is simply a peroxide of iron, and is exceedingly sensitive to oxygen. Hence on slight exposure to the atmosphere (as when the bottle remains unstopped,) it unites with the oxygen of the air, and the solid oxide of iron is formed. He suggests the following formula as one not generally known, for an antidote to arsenic, and claims for it precedence over all others; first, because it forms the surest antidote; and secondly, because the agents are almost always accessible—even to the country doctor who carries saddle bags:

℞. Muriatic tincture of iron................1 ounce.
Bicarbonate of soda (or potash).........1 ounce
Tepid water....................................Teacupful.
Mix.

The sesquioxide of iron is immediately formed in a solution of chloride of sodium (common salt.) Give this mixture almost *ad libitum.* It is a perfect antidote to arsenic.—*Vir. Medical Monthly.*

**Hydrobromate of Quinia as an Antipyretic.**—Dr. Esquerdo administered this salt to a typhoid patient, whose temperature had been 40° C. for several days; two days afterwards the temperature was reduced to 36° C. At this point, as the author feared collapse, since the reduction had been so considerable, the hydrobromate of quinia was no longer given; the temperature became higher. In two cases of phthisis the hydrobromate caused a disappearance of the feverish symp toms and swellings. The dominant action of hydrobromate of quinia consists in its power of causing a lowering of temperature and of diminishing the frequency of the pulse whilst it increases its amplitude. The observations of Dr. Esquerdo are corroborated by Dr. Cahis, who has observed in a typhoid patient a lowering of temperature from 41° to 39° or even to 38 C. brought on by the administration of fifty to seventy centigrammes (= 8 to 11 grains) of the hydrobromic salt.—*New Remedies.*

**Bismuth in Skin Affections.**—Dr. Sweet in Med. Summary, says: I wish briefly to call the attention of my medical brethren to the value of the subnitrate of bismuth as an *external* application. Whenever Erasmus Wilson recommends the oxide of zinc ointment, I use the bismuth, and with much more satisfactory results. I do not know what has been the experience of others, but I have found the zinc ungt. too stimulating for any acute eruptions. But the bismuth fulfills the indications perfectly. Mixed with cosmoline or fresh lard in almost any proportion, it is a sovereign remedy for eczema, herpes, intertrigo of infants, and anything where there is an abraded or irritated surface. A short time since I succeeded in healing an extensive ulcer of the leg, which had resisted other treatment. It is also an excellent application for piles, applied as an ungt. externally, or injected in the form of a solution—a teaspoonful to a few ounces of water or other fluid.

**Chloral in Retention of Urine.**—Dr. Tidd relates a case of twenty-four hours' retention of urine in a young woman in the eighth month of pregnancy. On account of tumefaction of the genital organs and some deviation of the urethra, all attempt at catheterism had failed.

Morphia was administered and puncture of the bladder proposed, when Dr. Tidd remembered the success which had attended the administration of chloral in like cases in the hands of some surgeons. He prescribed a solution of 10 grammes, (150 grains) of chloral in 60 grammes (℥ij) of water, and administered it in teaspoonful doses at first every half-hour, and then every two hours. Deep sleep ensued, in which the patient unconsciously passed an enormous quantity of urine. Excretion commenced five minutes after the second dose of the solution. Seven days later, natural labor occurred; child living and healthy; no recurrence of retention.—*Le Praticien.*

**Treatment of Carbuncle by Blisters.**—M. Jules Guerin, in a communication to the Academic de Medicine, says that the most efficacious mode of cutting short the progress of a carbuncle and hastening its cure is to cover the whole of the inflamed part with a large blister, having a hole in its center to admit of discharges. The blister must be continued on until complete vesication has taken place, and any portion of the carbuncle over which this has not done so will remain hard and resistant. When the blister has taken effect the pain is at once relieved, and the redness and resistance of the tumor disappears, and it becomes benign and inert, its enucleation proceeding under the use of ordinary means, without the aid of the bistoury. When after the discharge of its contents, a deep excavation remains, it is useful to apply to the walls a solution of nitrate of silver, with object of obliterating the open vascular orifices and impeding the absorption of the diseased liquid.—*Gas. des. Hop., Sept.* 14—*Cincinnati Clinic.*

**On the Prevention of Fatal Accidents from Using Anæsthetics.**—Dr. Simonin gives, in the Revue Med. de l' Est, the following three observations, which may be considered as very important if attended to: 1. Progressive peripheric insensibility, especially in the temporal region and the cornea. 2. The condition of the muscles and the jaws; the former must be in a complete state of relaxation, and the jaws closed. The adductor muscles of the lower jaw, therefore, form an exception to the rule, by being in a state of trismus. 3. The state of the pupil, which must be contracted, while the respiration becomes more normal, having been much quickened during the stage of excitement. All these phenomena are very important; they are synchronous, and must be carefully observed, as well as respiration and circulation. If the three symptoms cited should not appear coincidently they must be carefully watched for in various stages of the anæsthesia, because they are sure to appear at a given moment.—*Medical Reporter.*

**Antidote for Arsenite of Soda.**—The ordinary antidote to arsenious acid, hydrated sesquioxide of iron, is wholly inefficacious in poisoning by arsenite of soda or potassa. The antidote to the latter is formed by the mixture of solution of sesquichloride of iron and the oxide of magnesium. This mixture also answers for acid arsenious, consequently should always be preferred in arsenic-poisoning. Give the officinal sol. ferri sesquichlor., and afterward the magnesia. Give a cathartic an hour after the antidote. Avoid all acid drinks.—*New York Med. Record.*

**Ovariotomy Superseded.**—A proposal has been brought before the Paris Academy of Sciences by M. Tripier to establish a fistula between the cavity of an ovarian sac and the exterior. He has tried it in one case with success. The interior of the sac can in this way be washed out or treated with iodine injections or cauterized. He has used injections of iodized water daily. The galvano-caustic is used to establish the fistula. This operation is less formidable than ovariotomy, and can be easily carried out, but, of course, is not devoid of danger, but it may be applicable in cases where gastrotomy is refused or inapplicable. With regard to injections, they should not be too strong. We may point out that death from poisoning by iodine has been recorded where the drug was injected. This operation may be compared with electrolysis for ovarian dropsy.—*The Doctor*.

**Dichloride of Ethidene as an Anæsthetic.**—The Scientific Grants Committee of the British Medical Association have received from a special committee a report upon the action of this anæsthetic, in which it is claimed that dichloride of ethidene presents all the advantages of ether, without any of its disavantages; and that the following opinion of Steffen, given in Binz's *Evidences of Therapeutics*, is correct in most particulars: "It is said to have the following advantages over chloroform, which it resembles in its ultimate action, namely a pleasanter smell, the power of producing narcosis more rapidly, as well as without excitement or vomiting, more rapid recovery without after-effects, and altogether less danger."

In their experience, narcosis has not been produced more rapidly than with chloroform, but rapidity of narcosis depends very much on the mode of administration.—*Amer. Journal of Med. Sciences*.

**Salicylic Acid.**—M. A. Casson proposes, in the Bulletin General de Therapeutique, the employment of citrate of ammonia as a means of facilitating the solution of salicylic acid. Half a drachm of salicylic acid dissolves readily in less than four ounces of water (120 grammes) if 37 or 40 grains of citrate of ammonia are added. M. Casson gives the following formula: For a solution

| | | |
|---|---|---|
| ℞ | Salicylic acid | dr. i. |
| | Citrate of ammonia | dr. ss. |
| | Rum or brandy | oz. i. |
| | Distilled water | oz. v. |

A tablespoonful of this solution will contain from 4 to 4½ grains of salicylic acid. The citrate of ammonia is easily prepared by saturating ammonia in a solution of citric acid.

**Breech Presentations.**—Dr. T. Gaillard Thomas (Med. and Surg. Reporter), says in a clinic lecture:

Never hurry the early stages. Carefully refrain from bringing down a leg with the finger or blunt hook, but wait until the breech presses on the perineum; then turn the woman across the bed, and give a hypodermic injection of ergot. Remember the delivering force must come from above; have an assistant ready to press with all his force as soon as the cord can be reached, urging the woman to bear down with all her might. The result is usually a very speedy delivery, as soon as the finger can be got into the mouth.—*N. C. Med. Journal*.

**Relief of Pain from the Application of Sulphate of Copper.**—Dr. Pick, of Vienna, observes that it was by mere accident that he discovered the means of relieving the intense and enduring pain caused by the application of sulphate of copper in diseases of the conjunctiva. As in purulent ophthalmia these applications have sometimes to be made daily for months, the relief of such suffering is of great importance. The plan consists in sprinkling calomel over the parts to which the sulphate has been applied four or five minutes after they have been touched. The pain immediately diminishes; and after from three to six days of this procedure the calomel may be applied immediately after the touching with the caustic, and then the pain instantly disappears. —*Centralblatt.*

**Hiccough.**—Dr. Cochran, (Medical Brief,) had a case of hiccough with a case of chronic gastritis; gave assafœtida, valerian, ether and opium. Hiccough grew worse until third day, when patient could scarcely swallow. At suggestion of Dr. A. H. Scott, of Mansfield, I tried "circular compression of the base of the thorax," (Ziemssen's Cyclopedia, Vol. XI, page 339) by tight bandages, placing compress over apex of sternum, at same time gave morphia by subcutaneous injection. The hiccough was arrested within two minutes and did not return.

Why is not "circular compression of the base of the thorax," eminently practiced by relaxing, and thus "placing at rest" the diaphragm?

**Operation for Phimosis.**—M. Hart, of Rouen, reported the latest method in the London Medical Record. Eighty cases have already been operated in this way; among the patients were old men and children, and the appearance of the parts afterwards is said to be most elegant, there being no apparent scar. The prepuce on its dorsal aspect and opposite the base of the glans is pierced by a needle carrying a caoutchouc thread; the portion of the prepuce in front of the puncture is then ligatured and the operation is finished. After three or four days section is completed. The patients do not suffer and may, if necessary, continue their ordinary occupations.

**On the Action of Iodoform.**—Dr. Zeissl relates his experience of the remakably favorable results of the use of iodoform in venerial sores. He uses a powder for sprinkling the part, consisting of 7 centigrammes (little more than a grain) of iodoform in 5 grammes (75 grains) of sugar of milk. For internal use, he employs the following formula: iodoform, 1.5 gramme (22 grains); white sugar, 3 grammes (45 grains); to be divided into twenty powders of which one is taken thrice daily. He recommends this especially in the neuralgic affections of syphilis; it has been proved also very useful in certain cases of ordinary neuralgia. —*London Med. Record.*

**Ergot and Sodium Bromide in Epilepsy.**—Prof. Bauduy reports a case of epilepsy of 16 years standing, which was cured by giving twenty grains of bromide of sodium, with half a drachm of fluid extract of ergot three times a day. This treatment was continued a year and a half, and four years have elapsed without the recurrence of a fit.—*Lancet and Clinic.*

**Can One Have Typhoid Fever After Forty?**—A correspondent of the Lancet puts these hard questions:

"Can it be seriously conceived that a man can possibly have *typhoid fever* (as people will erroneously call it) after he has passed the age of susceptibility to its influence?—in other words, when he has lived so long as to have nothing of Peyer's patches left in him?"

The thing is physically impossible: and if, after the age of forty, cases really crop up, the time has arrived for framing another new and sounder pathology of enteric fever than the generally received one of to-day. Now, then, accepting current pathology of the subject as infallible, the question arises, why should forty be singled out as the limited period of liability, and the succeeding years past the prime of life as the period of immunity? The reason is simple. It is this: Forty years is the time of life when Peyer's patches (the special seat of lesion) begin to degenerate, and enteric fever cannot touch the advanced in years, because Peyer's glands are absent in the old. *Ergo* I am at liberty to question the diagnosis "typhoid fever" in such an individual.—*Detroit Lancet.*

**Condition of the Tongue Valuable in the Diagnosis of Gastric Disorders.**—Dr. Wilson Fox gives, as valuable aids in the diagnosis of gastric disorders, the following conditions of the tongue:

1st. Dyspepsia, with distinct atony of the stomach. The tongue broad, pale and flabby, the papillæ generally enlarged, more especially at the tip and edges.

2d. Dyspepsia from irritative causes. The tongue is redder than usual; often of a bright, florid color, or even raw-looking. It is often pointed at the tip, which, together with the sides, presents an extreme degree of injection, the papillæ standing out as vivid red points. This form is often associated with aphthæ, and is most common in scrofulous children and phthisical adults.

3d. Dyspepsia from excessive or hurried eating is apt to present a tongue uniformly covered through the greater part of its surface with a thick fur, whitish or brownish, with some degree of enlargement and redness of the papillæ at the tip and edges.

4th. Neuroses of the stomach display a tongue which, as a rule, is clean, though often pale, broad and flabby.—*Lancet.*

**The Differential Diagnosis between true Epilepsy and Hystero-Epilepsy.**—M. Charcot (Gazette des Hopitaux, 1878, No. 49), says that true epilepsy develops itself, after only a short aura, in the form of tonic and clonic spasms accompanied by marked stertor.—The convulsive stage of the hysterical paroxysm is preceded, after an aura lasting one, two, or even several days, by a peculiar, prolonged cry; this is followed by violent, purposeless, fantastic movements, clonic spasms, and great physical excitement, perhaps even delirium; but none of these symptoms are accompanied by the slightest signs of stertor.—*London Med. Record.*

**Carbolic Acid in Nasal Polypus.**—Dr. J. A. Henning reports a case of nasal polypus, in which he injected twenty drops of a mixture of carbolic acid, one part, and glycerine, four parts. In a month every trace of the polypus had disappeared.—*Brief.*

**Belladonna Hypodermically in Congestive Chills.**—Dr. T. W. Rankin highly recommends the hypodermic use of belladonna in congestive chills, not only as a valuable adjunct, but even as the *prime remedy*. He relates the record of four cases as best exemplifying the type of the disease and his method of treatment. The patients presented about the following symptoms: Loss of consciousness, no pulse at the wrist; face, trunk and extremities moist and cold; breathing stertorious and superficial; eyes glassy and pupils not responding to light, etc. He administers tincture of belladonna in ten-drop doses hypodermically at short intervals, until the pulse is felt at the wrist. Before the return of another paroxysm, he gives large doses of quinine per os, as in any severe intermittent.—*Detroit Lancet.*

**Pyrogallic Acid in Psoriasis.**—Dr. A. Jarisch (Pharmaceutische Post), reports his complete success in the treatment of psoriasis by pyrogallic acid. At first he used an ointment, containing 20 per cent. of pyrogallic acid; this was, however, found to produce excoriations. Hence he has reduced the ointment, as ordinarily used, to the strength of 10 per cent., and in some cases he uses it only of 5 per cent. If spread on muslin, and then applied, it must be still further diluted, otherwise it acts as an irritant. Aqueous solutions should contain about 1 per cent. Pyrogallic acid acts not as rapidly as chrysophanic acid, but it is equally certain in its results.—*London Medical Record, Dec.* 15, 1878.

**Ergotine in Ophthalmia.**—Dr. Planat, in Journal de Therapeutique, states that in purely inflammatory diseases of the eye, he has obtained great benefit from the use of ergotine. Twenty grains of ergotine are dissolved in five drachms of rose water, or glycerine, and every two hours from eight to ten drops are inserted in the eye. If there be violent inflammation of the eyelids, a cloth wet with the solution is left on the parts for some hours. In two or three days he is thus able to subdue cases of most intense blephero-conjunctivitis. In inflammations of the cornea, it is of less service, though still of positive value. In iritis, it is of great service in rapidly subduing the acute manifestations and preventing their extension to the external membranes of the eye.—*Detroit Lancet.*

**Cataract.**—In the Gazette des Hopitaux, January 16, 1879, Dr. Tamanichef reports a case of a man, 48 years of age, of a strong plethoric constitution, who had a cortical cataract of the left eye, for which was prescribed protoiodide of mercury, which he took for a month and a half with good results. The treatment interfering with the patient's occupation, it was discontinued, and iodide of potassium was given internally. Three or four months of this treatment was continued, when the patient began to read large typographical characters, but died suddenly, from apoplectic seizures. This and other well known cases, the author considers, prove the absorption of the opacified crystalline in cases of cortical cataract. In explanation of this mode of treatment, the author states that the remedies pass through the general circulation into the liquids of the eye, and operate immediately upon the inflammatory process of the crystalline.

